# Polymeric Kinetic Hydrate Inhibitors in NaCl Brine–Sediment Media: Hydrate Performance, Rheological Structuring, and Interfacial Responses

**DOI:** 10.3390/gels12090842

**Published:** 2026-09-15

**Authors:** Guan-Lin Zhong, Ren Wang, Jian-Long Wang, Hui-Cui Sun, Jin-Sheng Sun, Lu-Man Liu, Jin-Tao Weng, Ping-Ya Luo

**Affiliations:** 1 School of Oil & Natural Gas Engineering, Southwest Petroleum University, Chengdu 610500, China; zhonggl000@163.com (G.-L.Z.);; 2 CNPC Engineering Technology R&D Company Limited, Beijing 102206, China; 3 International Exchange and Cooperation Office, China University of Geosciences (Beijing), Beijing 100083, China

**Keywords:** natural gas hydrate, kinetic hydrate inhibitor, polymer–particle association, rheology, viscoelasticity, 3.5 wt% NaCl brine, sediment interface, structural recovery

## Abstract

Gas hydrate plugging remains a persistent flow-assurance challenge in offshore oil and gas operations. PVP, pectin, CMCS, and PASP were compared at 0.1–0.5 wt% in pure water, 3.5 wt% NaCl brine, pure water–sediment, and brine–sediment media using hydrate kinetics, Raman spectroscopy, CST, SEM/EDS, zeta potential, viscosity screening, and quantitative rheology. At 0.5 wt% and 3 °C, all sediment-containing formulations were shear-thinning. CSS ramps revealed continuous stress-dependent flow consistent with progressive yielding, while oscillatory measurements indicated weak elastic-dominant responses for polymer-containing suspensions. Pectin gave the highest storage modulus and the strongest salinity-induced increase in oscillatory flow-point stress (approximately 0.092 to 0.178 Pa), whereas PASP retained the most favorable multimetric KHI response and the highest 500 s recovery in brine–sediment. PVP shifted from an oscillatory flow point above 0.209 Pa in pure water–sediment to approximately 0.156 Pa in brine–sediment. These results show that small-deformation stiffness, steady yielding, oscillatory flow transition, post-shear recovery, and KHI performance are distinct but partially coupled material responses. The combined evidence supports weak, deformable polymer–sediment structuring rather than a single rheological strength mechanism governing hydrate inhibition.

## 1. Introduction

Natural gas hydrates (NGHs) are ice-like crystalline inclusion compounds in which water molecules form hydrogen-bonded cage structures that encapsulate methane or other guest molecules under high-pressure and low-temperature conditions. They are widely distributed in deep-sea sediments and permafrost regions [1,2]. During offshore oil and gas drilling, production, and transportation, however, uncontrolled NGH formation, deposition, and agglomeration can cause severe blockage in wellbores and pipelines, making hydrate management a central flow-assurance problem [3,4]. Hydrate inhibitors used in industry are generally divided into thermodynamic hydrate inhibitors (THIs) and kinetic hydrate inhibitors (KHIs) [5,6]. Conventional THIs such as methanol and ethylene glycol reduce hydrate risk by shifting phase-equilibrium conditions, but they require large dosages, increase operating cost, and raise environmental concerns because of their volatility and toxicity [7,8]. KHIs can delay hydrate nucleation and/or hinder crystal growth at relatively low dosages, making them more attractive for low-carbon and environmentally constrained flow-assurance scenarios [9,10]. However, traditional commercial KHIs such as polyvinylpyrrolidone (PVP) and poly(N-vinylcaprolactam) (PVCap) have limited biodegradability and may pose long-term ecological risks in marine environments [11,12].

Natural or bio-based polymers such as pectin [13,14], amino acid-derived materials [15,16], and chitosan derivatives [17,18] have been explored as KHI candidates, with selected formulations reported to offer favorable biodegradation or environmental profiles [19]. These reported environmental attributes provide context for candidate selection. Such polymers may influence hydrate formation through water binding, perturbation of water structure, and modulation of interfacial mass transfer [15,20,21]. PVP was included as a widely studied laboratory benchmark; PVCap-based formulations remain a higher-performance commercial reference [6,9,12].

Previous pectin studies also demonstrate that KHI ranking depends strongly on test protocol [22]. Xu et al. reported pronounced anti-nucleation and crystal growth effects in induction time and crystal growth inhibition tests [14], whereas Aminnaji et al. observed limited seeded crystal growth inhibition and emphasized the importance of hydrate history control [13]. Water-soluble poly(amino acid)s, including homo-poly(aspartic acid), have also been evaluated by slow constant cooling rather than the fixed-temperature induction protocol used here [23]. Direct numerical ranking across these studies is inappropriate because pressure, subcooling, gas composition, polymer source/molecular weight, seeding history, and endpoint definition differ. The present study therefore focuses on within-study comparisons across four media under one reactor protocol, with a direct contextual comparison to the representative pectin and poly(aspartic acid) literature provided in Section 2.5.

Dilute polymer–sediment suspensions can couple hydration, particle association, electrostatic interactions, viscoelasticity, yielding, and structural recovery. A single apparent viscosity value cannot resolve these contributions. Accordingly, the mechanical state of the present flowable suspensions was characterized by steady shear, controlled-shear-stress (CSS) ramps, oscillatory amplitude/frequency sweeps, and post-shear recovery. The CSS ramp and the oscillatory G′ = G″ flow point probe complementary stages of stress-induced structural weakening and flow [24,25,26].

Here, PVP, pectin, CMCS, and PASP were compared in pure water, 3.5 wt% NaCl brine, pure water–sediment, and 3.5 wt% NaCl brine–sediment under one reactor protocol. Hydrate kinetics were integrated with Raman, CST, SEM/EDS, zeta potential, viscosity screening, and quantitative rheology to resolve medium-dependent performance and test whether stronger sediment-associated rheological structuring necessarily accompanies stronger KHI performance.

## 2. Results and Discussion

### 2.1. Hydrate Inhibition Performance

**Figure 1:**
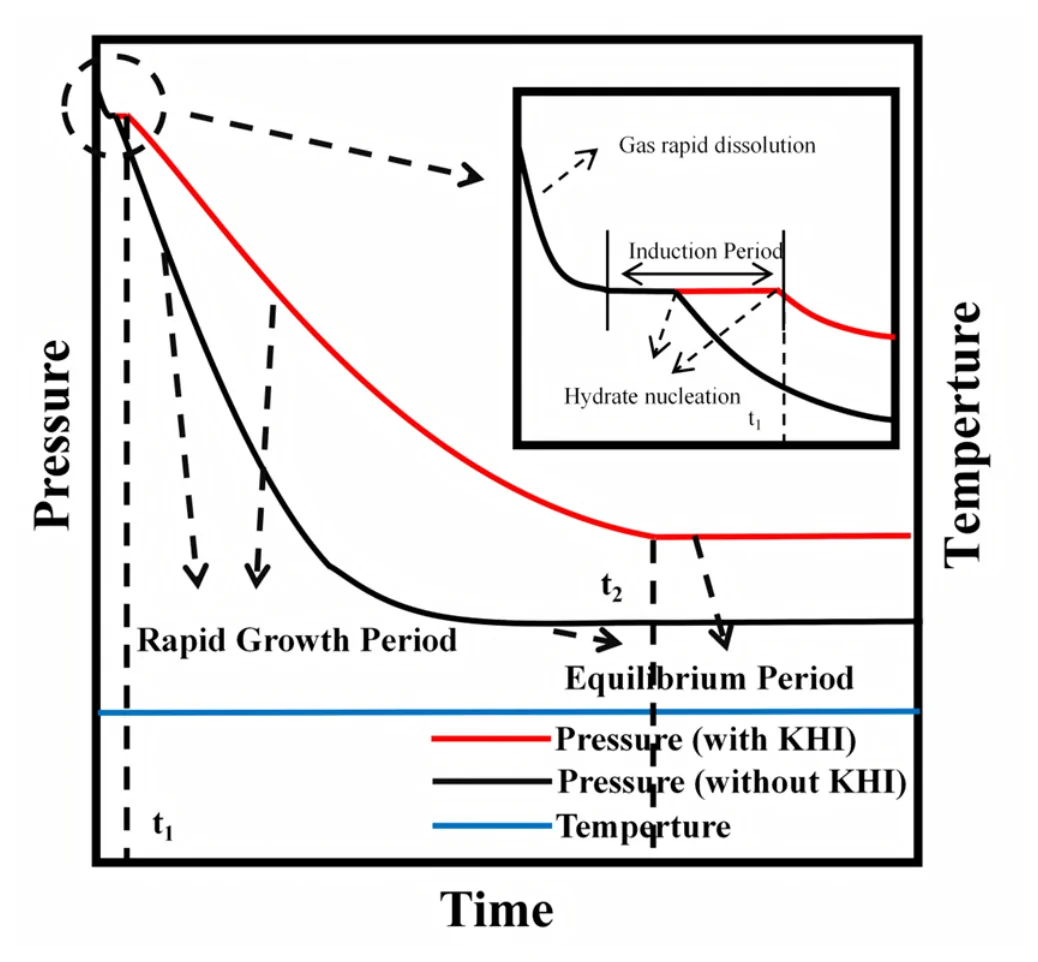
Representative pressure–temperature curves and the operational definition of methane hydrate formation stages.

Pressure–temperature profiles were divided operationally into gas dissolution, induction, rapid growth, and approach to equilibrium (Figure 1). The induction point was identified from the onset of a sustained pressure decrease and/or exothermic temperature signal. Because KHIs can affect nucleation, growth, and interfacial transport simultaneously, performance was evaluated from coupled kinetic descriptors rather than a single endpoint.

**Figure 2:**
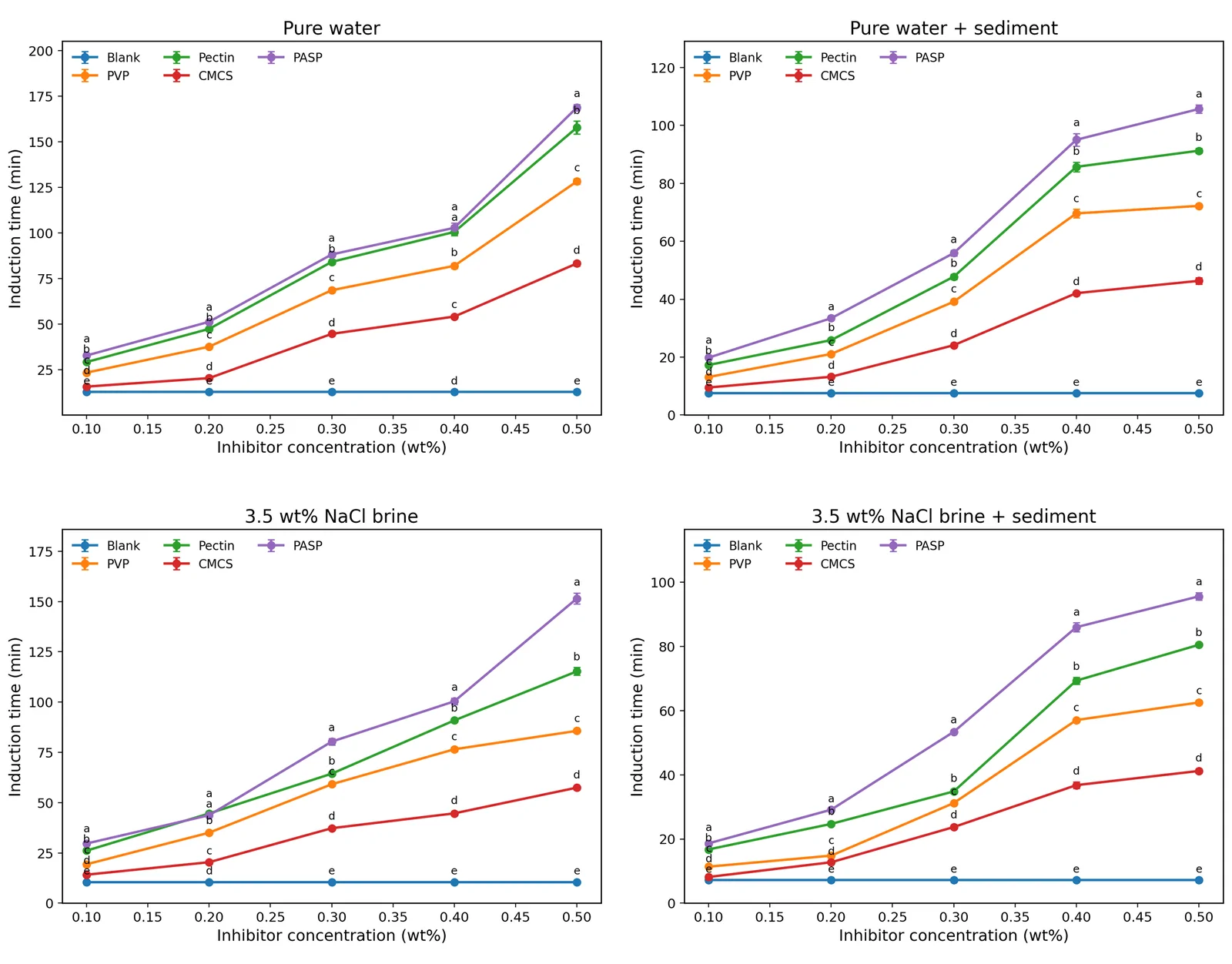
Effects of inhibitor concentration and medium on methane hydrate induction time. Error bars denote SD (*n* = 3). Within each concentration and medium, formulations not sharing a lowercase letter differ significantly at adjusted *p* < 0.05.

Induction time increased with inhibitor dosage in all media (Figure 2). In pure water, PASP increased from 32.80 min at 0.1 wt% to 168.75 min at 0.5 wt%, compared with 12.90 min for the blank. Sediment shortened induction more strongly than NaCl alone for both the blank and 0.5 wt% PASP under the present protocol. At 0.5 wt%, formulation effects were significant in all four media (*p* < 0.001), with PASP giving the longest induction time.

**Figure 3:**
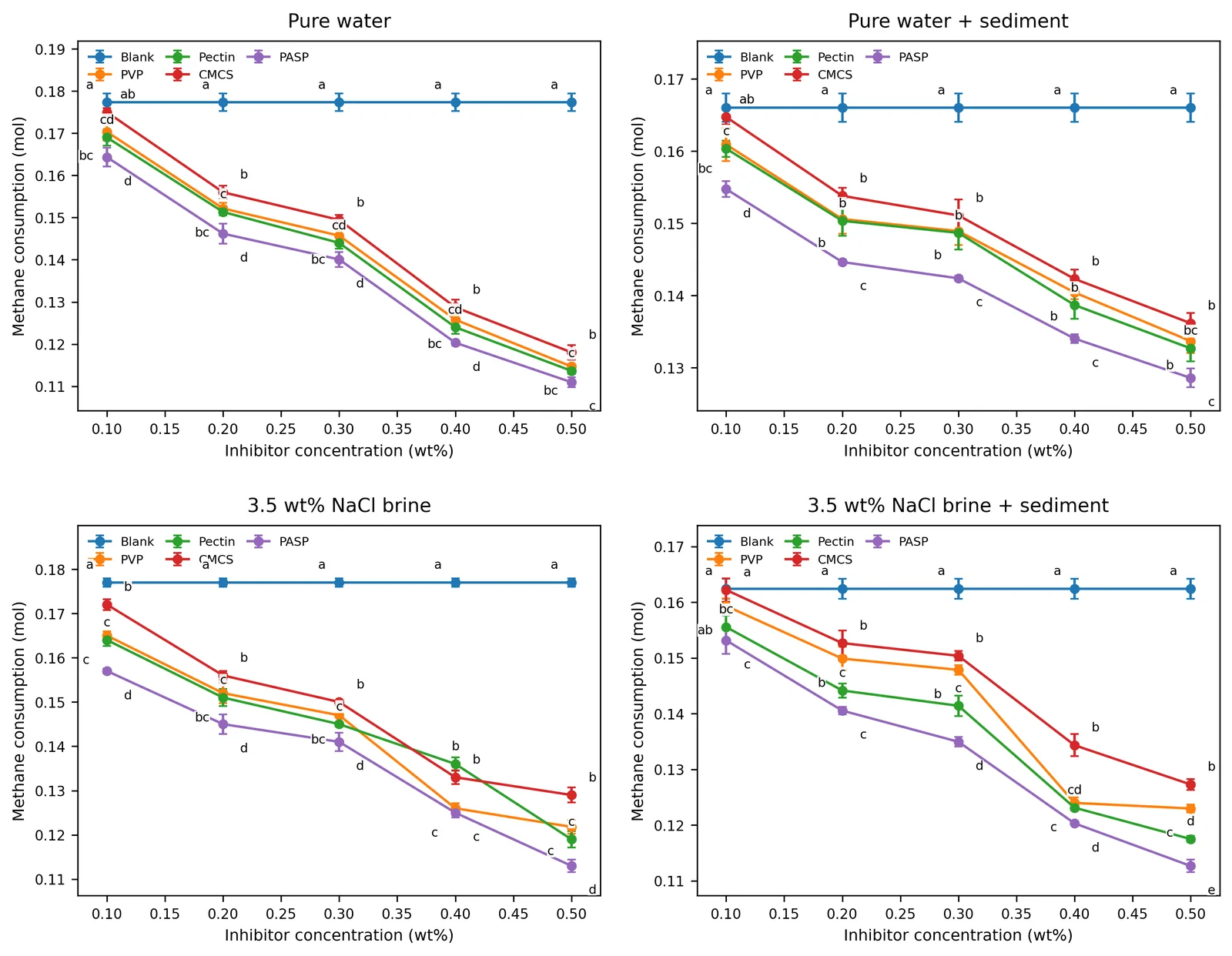
Effects of inhibitor concentration and medium on cumulative methane consumption. Error bars denote SD (*n* = 3). Within each concentration and medium, formulations not sharing a lowercase letter differ significantly at adjusted *p* < 0.05.

**Figure 4:**
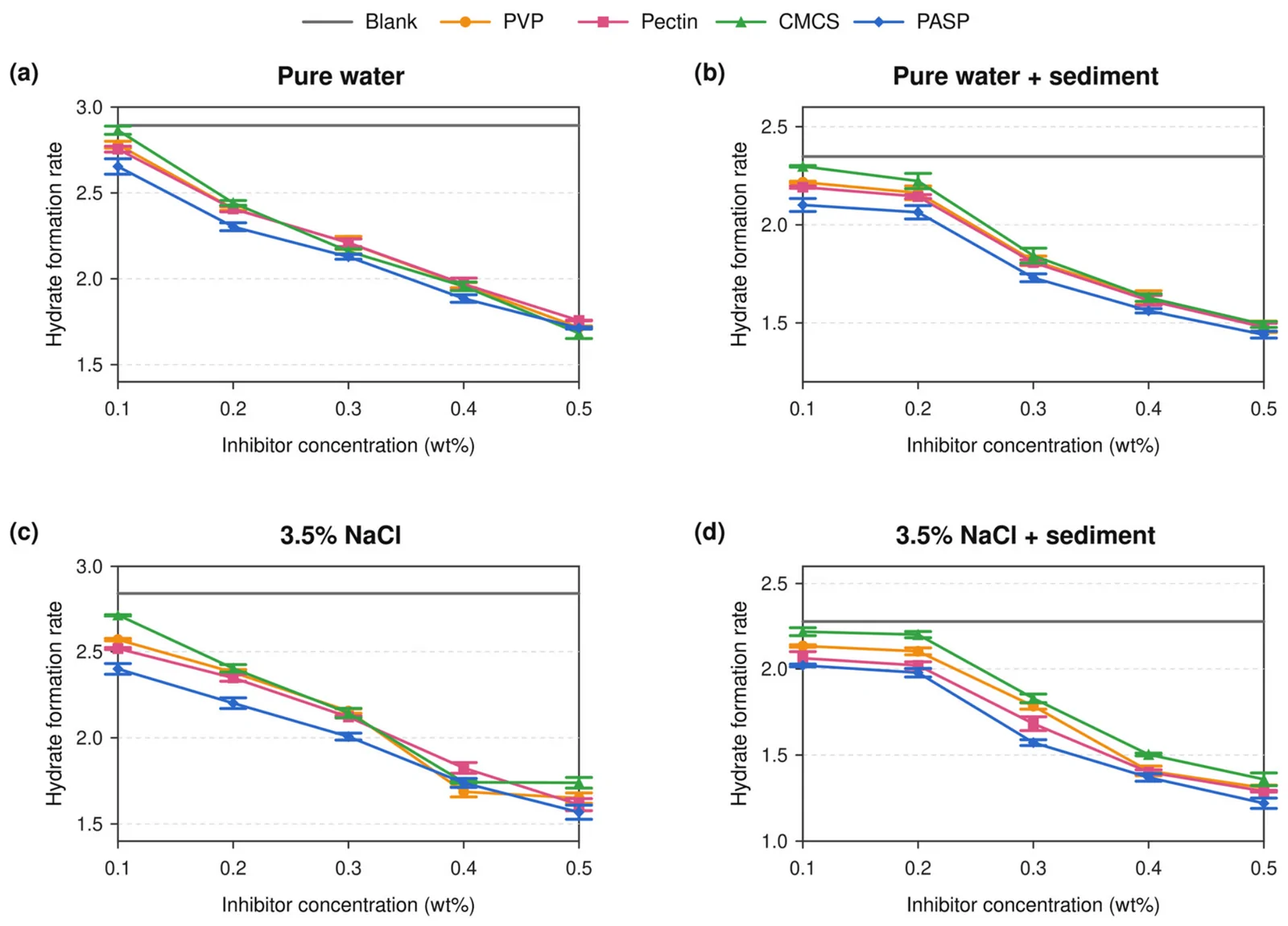
Effects of inhibitor concentration and medium on average methane-consumption rate: (**a**) pure water; (**b**) pure water–sediment; (**c**) 3.5 wt% NaCl brine; and (**d**) 3.5 wt% NaCl brine–sediment. Error bars denote SD (*n* = 3).

Methane consumption and average formation rate decreased with increasing inhibitor concentration (Figure 3 and Figure 4). In pure water, increasing PASP from 0.1 to 0.5 wt% lowered methane consumption from 0.164 to 0.111 mol and the average formation rate from 2.654 × 10^−4^ to 1.713 × 10^−4^ mol/min. In brine–sediment at 0.5 wt%, PASP retained the lowest methane consumption (0.113 mol) and average formation rate (1.220 × 10^−4^ mol/min). Sediment altered induction and growth differently, consistent with coupled effects on heterogeneous nucleation, water availability, mixing, and transport.

**Figure 5:**
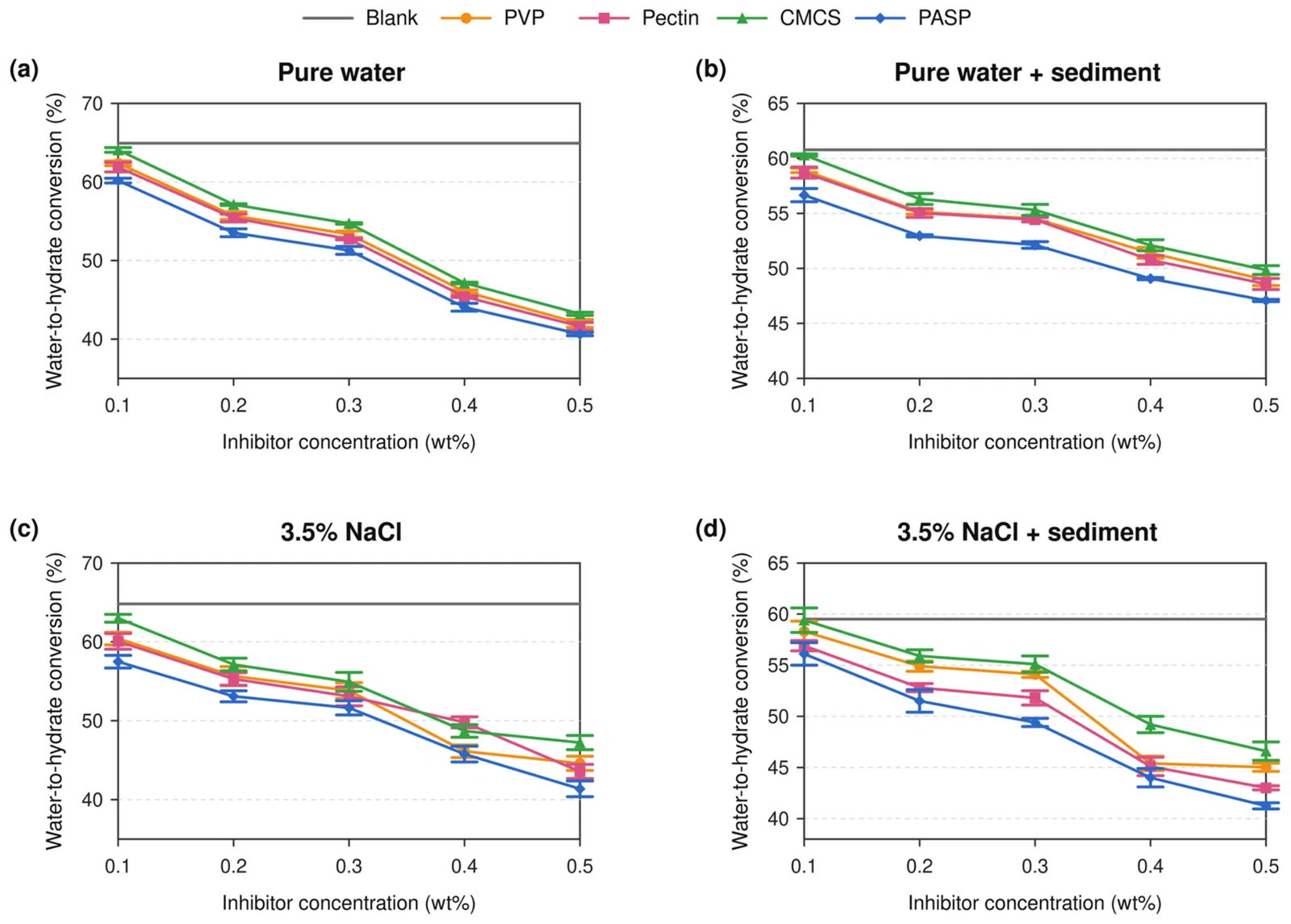
Effects of inhibitor concentration and medium on water-normalized apparent hydrate conversion: (**a**) pure water; (**b**) pure water–sediment; (**c**) 3.5 wt% NaCl brine; and (**d**) 3.5 wt% NaCl brine–sediment. Error bars denote SD (*n* = 3).

Water-normalized apparent conversion also decreased with inhibitor concentration (Figure 5). At 0.5 wt% in pure water, PASP, pectin, and PVP gave similar low conversions (40.63–41.99%), whereas CMCS remained higher (50.65%). Sediment changed the blank and PASP responses in opposite directions, reinforcing that medium effects cannot be represented by induction time alone.

Across induction, methane consumption, formation rate, and apparent conversion, PASP and pectin were the most favorable overall, PVP was intermediate, and CMCS was weaker within the tested window. The exact ordering remained descriptor- and medium-dependent, establishing the need for multimetric rather than single-endpoint assessment.

### 2.2. Exploratory Integrated Performance Evaluation

An exploratory normalized performance index (NPI) summarized the 0.5 wt% dataset using equal weighting within each medium. Induction time was treated as favorable when larger, whereas methane consumption, formation rate, and apparent conversion were favorable when smaller; sediment-specific scores additionally included |zeta potential| and apparent viscosity. PASP gave the highest common-descriptor NPI across all four media, while pectin showed comparatively stable scores, particularly in NaCl-containing systems (Table 1). The NPI is used only as a within-study screening summary.

### 2.3. Water-Phase and Sediment Interface Responses

Raman C, CST, freeze-dried SEM/EDS, and zeta potential were used as complementary descriptors of water-phase, migration, electrokinetic, and ex situ interfacial responses. Their convergence can support a mechanistic interpretation, but no single descriptor is treated as direct evidence of hydrate surface adsorption or a percolated gel network.

**Figure 6:**
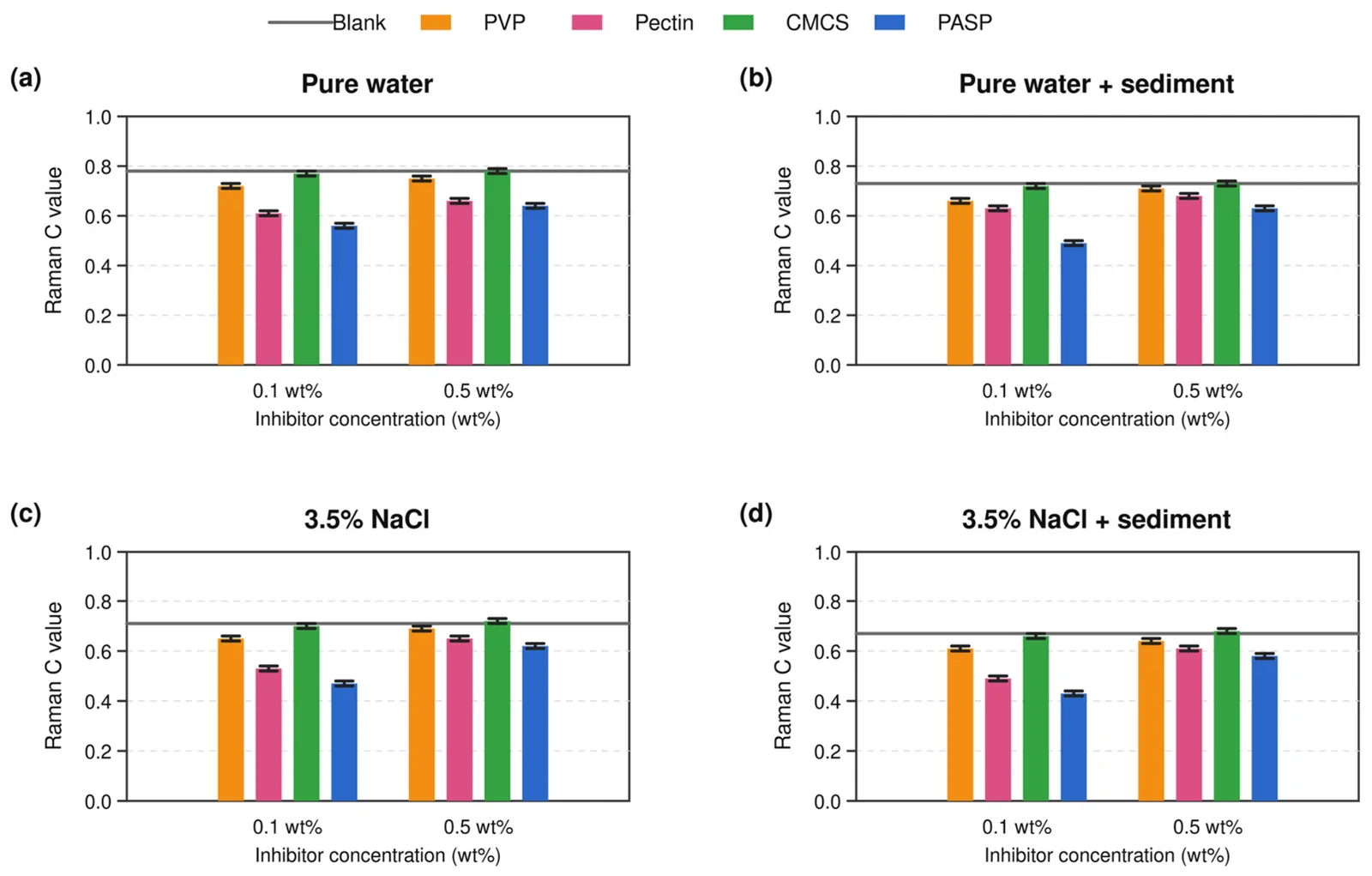
Effects of inhibitors on the Raman relative intensity C in the O–H stretching region: (**a**) pure water; (**b**) pure water–sediment; (**c**) 3.5 wt% NaCl brine; and (**d**) 3.5 wt% NaCl brine–sediment. Error bars denote SD (*n* = 3).

Raman C compared the fitted hydrogen bonding states in the O–H stretching region [27,28]. PASP and pectin produced larger C-value changes than PVP, whereas CMCS showed a weaker response (Figure 6). These differences are consistent with polymer-dependent hydration and hydrogen bonding and are interpreted as bulk water-phase evidence rather than direct hydrate-cage or hydrate-interface measurements.

**Figure 7:**
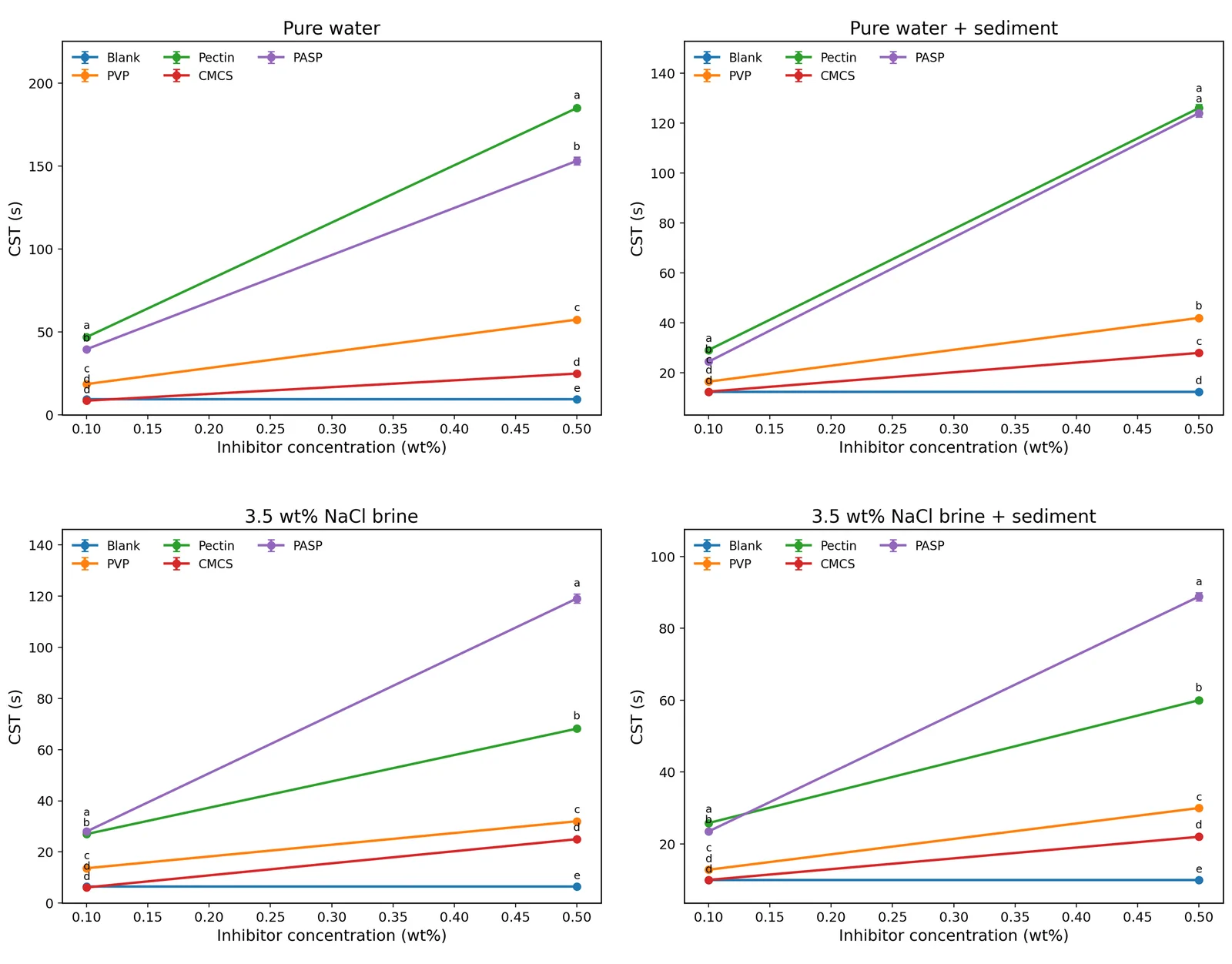
Effects of inhibitor concentration and medium on capillary suction time (CST). Error bars denote SD (*n* = 3). Within each concentration and medium, formulations not sharing a lowercase letter differ significantly at adjusted *p* < 0.05.

CST increased most strongly for pectin and PASP (Figure 7), indicating slower liquid migration through the test geometry [29]. At 0.5 wt%, formulation effects were significant in all four media (*p* < 0.001); pectin and PASP were statistically comparable in pure water–sediment, whereas PASP ranked above pectin, PVP, CMCS, and the blank in brine–sediment. CST is interpreted together with viscosity and particle response data because hydration, viscosity, and particle association can contribute simultaneously.

**Figure 8:**
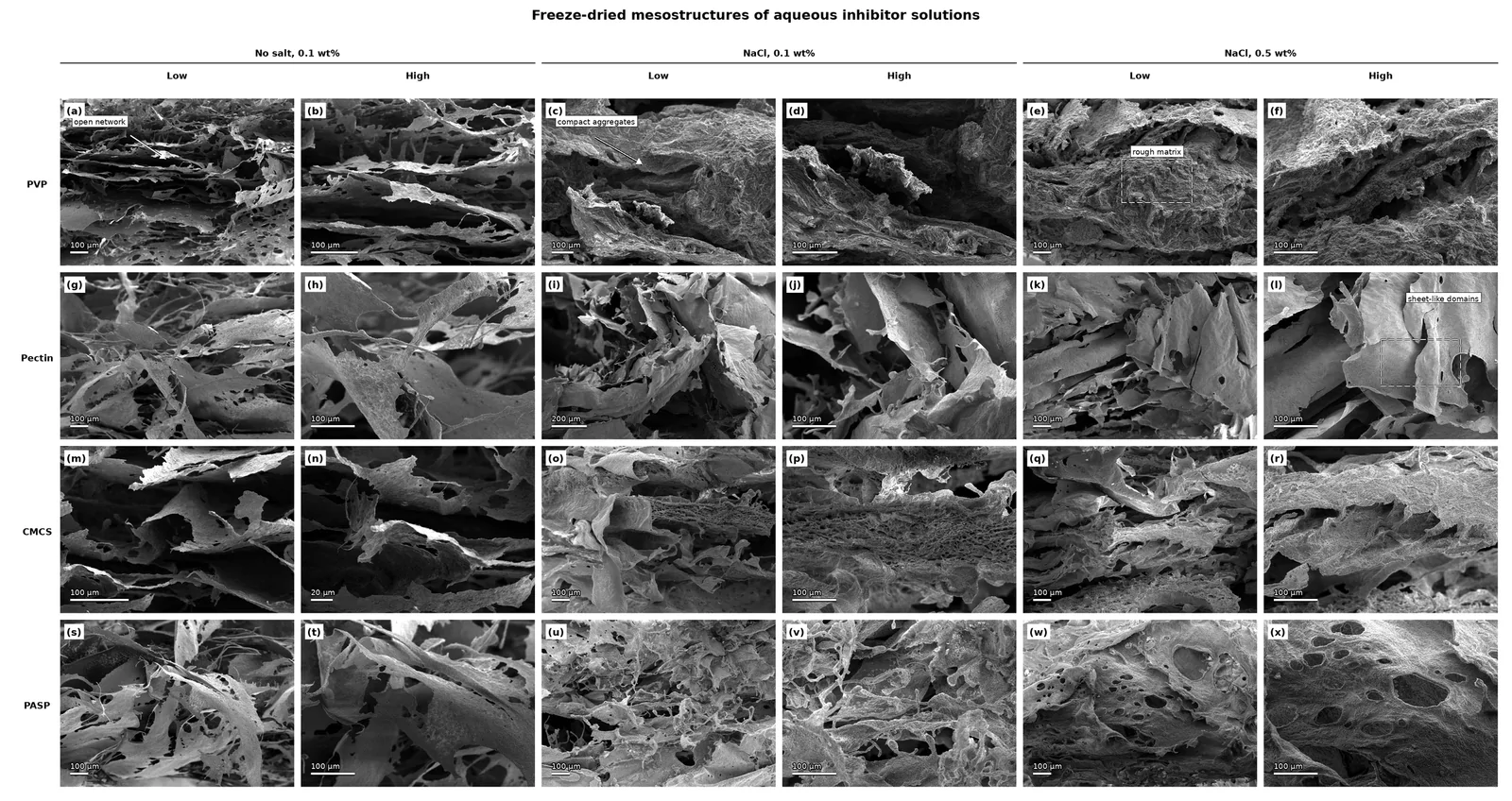
SEM morphologies of freeze-dried inhibitor solutions: (**a**,**b**) PVP, no salt, 0.1 wt%, low/high magnification; (**c**,**d**) PVP, NaCl, 0.1 wt%, low/high; (**e**,**f**) PVP, NaCl, 0.5 wt%, low/high; (**g**,**h**) pectin, no salt, 0.1 wt%, low/high; (**i**,**j**) pectin, NaCl, 0.1 wt%, low/high; (**k**,**l**) pectin, NaCl, 0.5 wt%, low/high; (**m**,**n**) CMCS, no salt, 0.1 wt%, low/high; (**o**,**p**) CMCS, NaCl, 0.1 wt%, low/high; (**q**,**r**) CMCS, NaCl, 0.5 wt%, low/high; (**s**,**t**) PASP, no salt, 0.1 wt%, low/high; (**u**,**v**) PASP, NaCl, 0.1 wt%, low/high; and (**w**,**x**) PASP, NaCl, 0.5 wt%, low/high.

**Figure 9:**
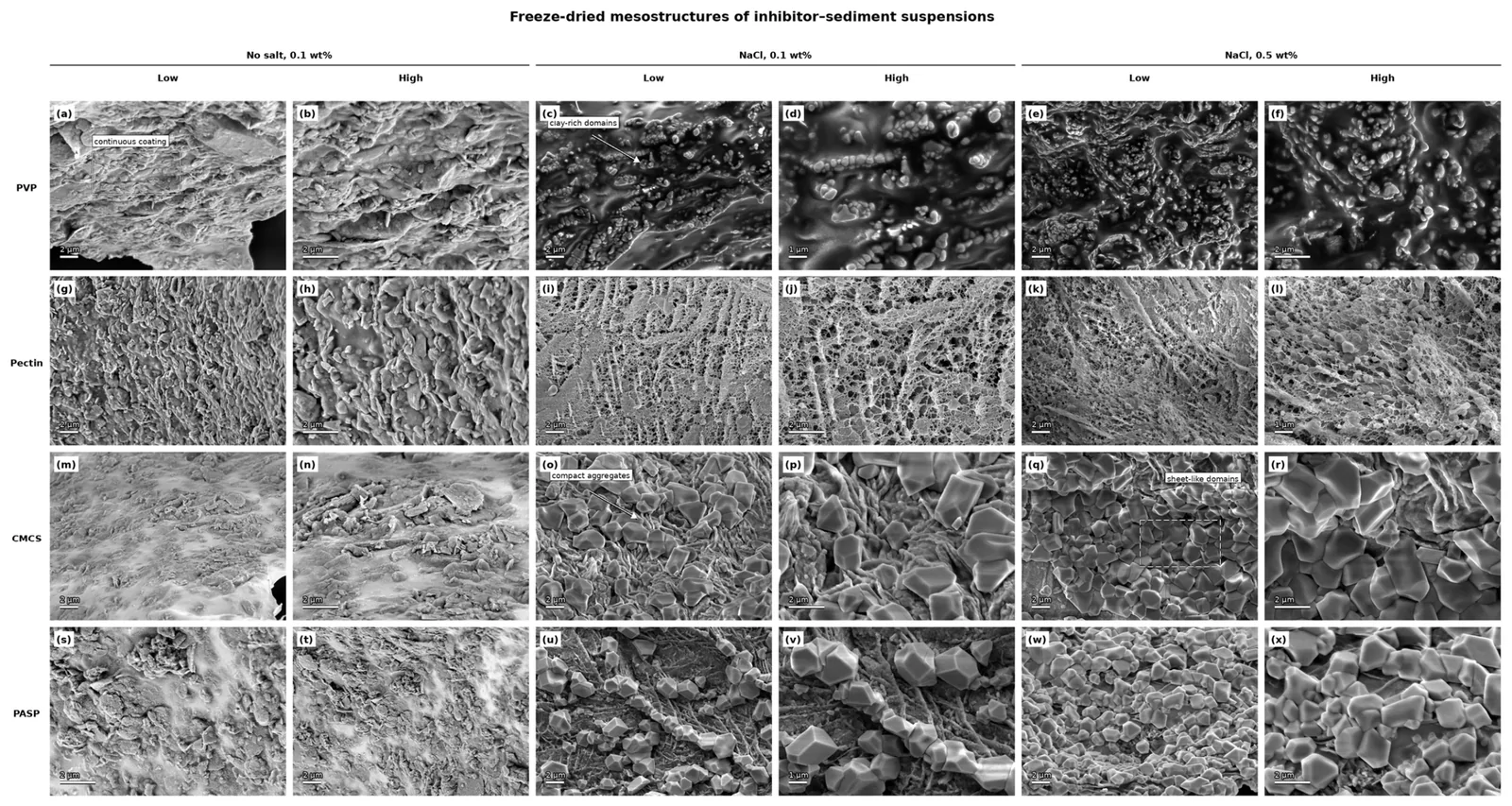
SEM morphologies of freeze-dried inhibitor–sediment suspensions: (**a**,**b**) PVP, no salt, 0.1 wt%, low/high magnification; (**c**,**d**) PVP, NaCl, 0.1 wt%, low/high; (**e**,**f**) PVP, NaCl, 0.5 wt%, low/high; (**g**,**h**) pectin, no salt, 0.1 wt%, low/high; (**i**,**j**) pectin, NaCl, 0.1 wt%, low/high; (**k**,**l**) pectin, NaCl, 0.5 wt%, low/high; (**m**,**n**) CMCS, no salt, 0.1 wt%, low/high; (**o**,**p**) CMCS, NaCl, 0.1 wt%, low/high; (**q**,**r**) CMCS, NaCl, 0.5 wt%, low/high; (**s**,**t**) PASP, no salt, 0.1 wt%, low/high; (**u**,**v**) PASP, NaCl, 0.1 wt%, low/high; and (**w**,**x**) PASP, NaCl, 0.5 wt%, low/high.

Freeze-dried SEM revealed polymer- and salinity-dependent mesostructures (Figure 8 and Figure 9). Salt-free samples were generally more open and sheet-like, whereas NaCl-containing samples showed more compact, wrinkled, or aggregated regions; sediment-containing filter cakes additionally displayed mineral-associated and coating-like domains. These images are treated as ex situ morphology because filtration, freezing, drying, and local sampling can reshape the observed structure.

**Figure 10:**
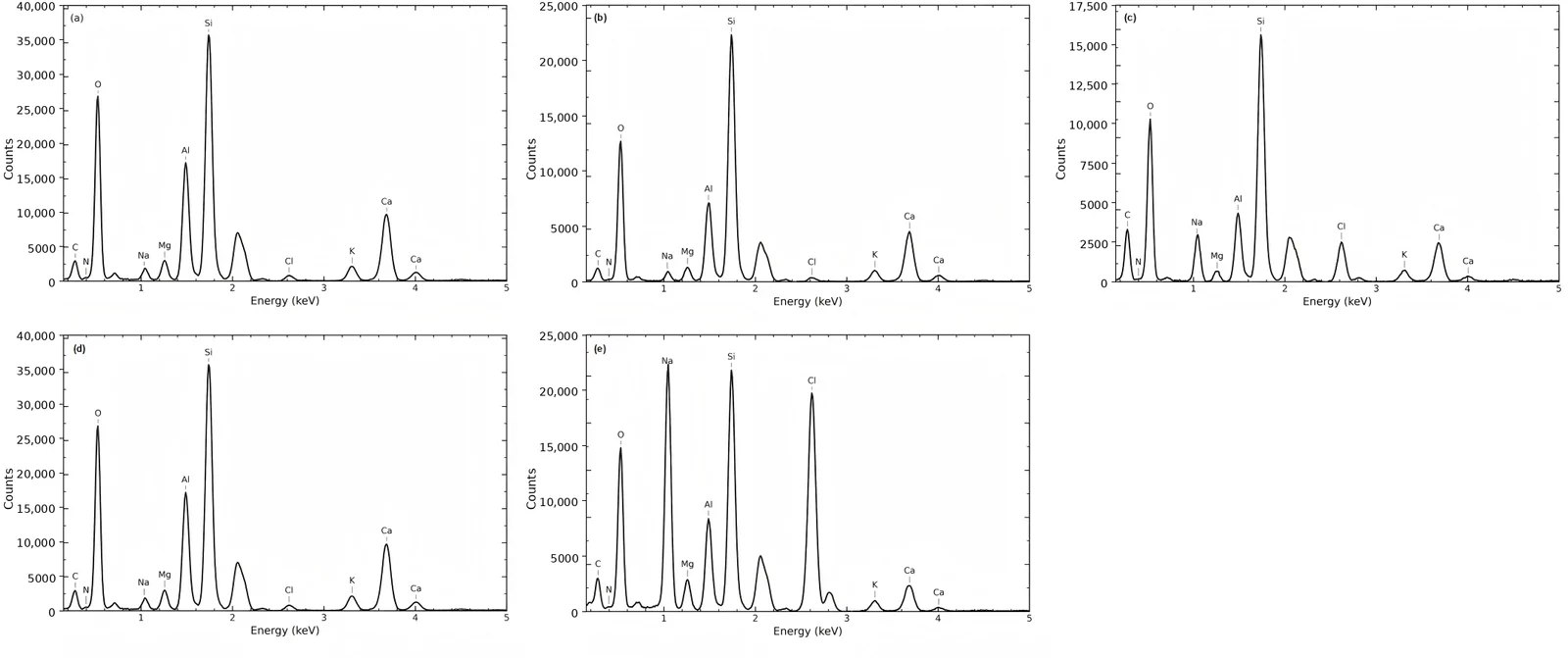
EDS mapping of representative freeze-dried filter-cake regions: (**a**) sediment; (**b**) sediment + 0.5 wt% PVP; (**c**) sediment + 0.5 wt% pectin; (**d**) sediment + 0.5 wt% CMCS; (**e**) sediment + 0.5 wt% PASP.

The sediment contained 33.2 wt% clay minerals, with illite dominant within the clay fraction, and D50 values of 8.81 and 8.68 μm. EDS of representative filter-cake regions showed polymer-dependent differences in C, Na, Cl, Al, Si, and Ca (Table 2; Figure 10), consistent with different polymer/mineral/salt associations at the dried surface. Together with Raman and CST, these results define distinct water-phase and sediment-associated responses without assigning a unique molecular pathway.

### 2.4. Suspension Electrokinetics and Rheological Structuring

**Figure 11:**
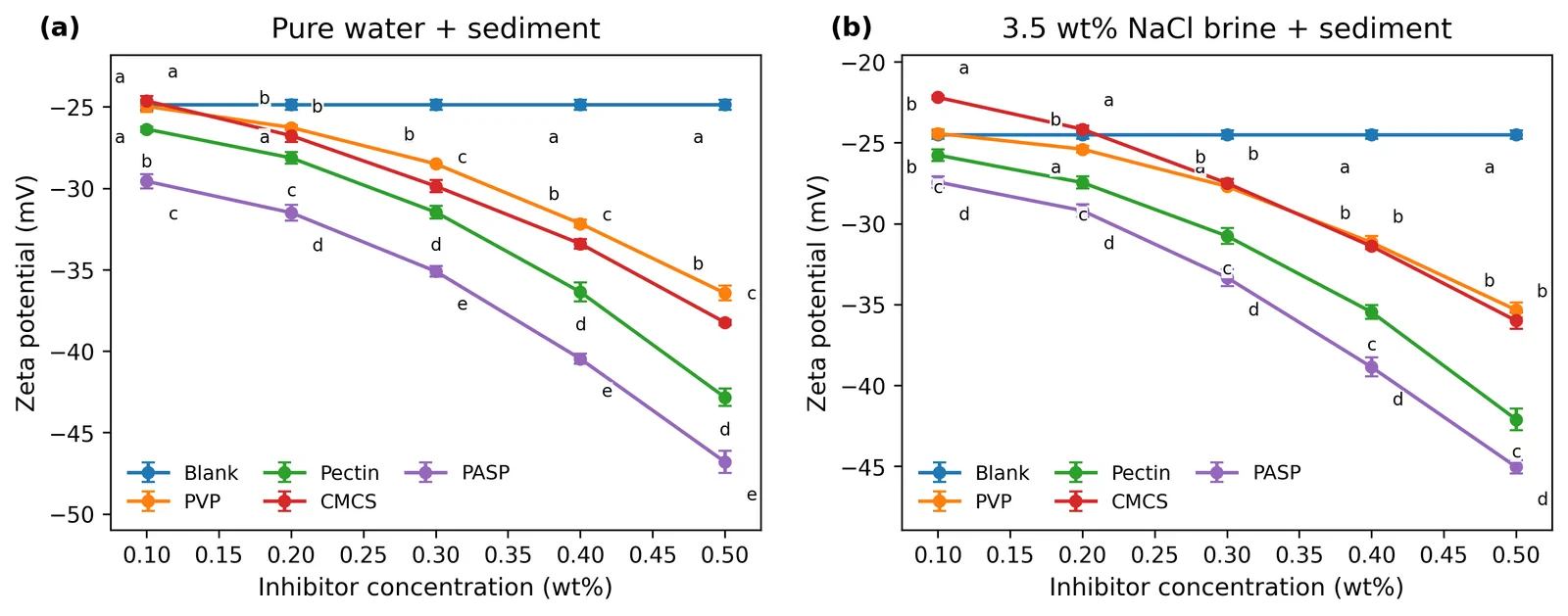
Effects of inhibitor concentration on the zeta potential of sediment suspensions: (**a**) pure water–sediment; and (**b**) 3.5 wt% NaCl brine–sediment. Error bars denote SD (*n* = 3). Within each concentration and medium, formulations not sharing a lowercase letter differ significantly at adjusted *p* < 0.05 according to the variance-adaptive procedure in Section 4.2.8.

Zeta potential quantified sediment suspension electrokinetics [30], while rheology resolved bulk flow and viscoelastic structure. At 0.5 wt% in pure water–sediment, PASP, pectin, CMCS, and PVP shifted the zeta potential from −24.9 mV for the blank to −46.8, −42.8, −38.2, and −36.4 mV, respectively (Figure 11). Significant formulation effects persisted in both sediment-containing media (*p* < 0.001), with PASP showing the most negative response. The ordering supports polymer-dependent changes in suspension electrostatics but is not assumed to determine KHI performance.

#### 2.4.1. Apparent Viscosity Screening

**Figure 12:**
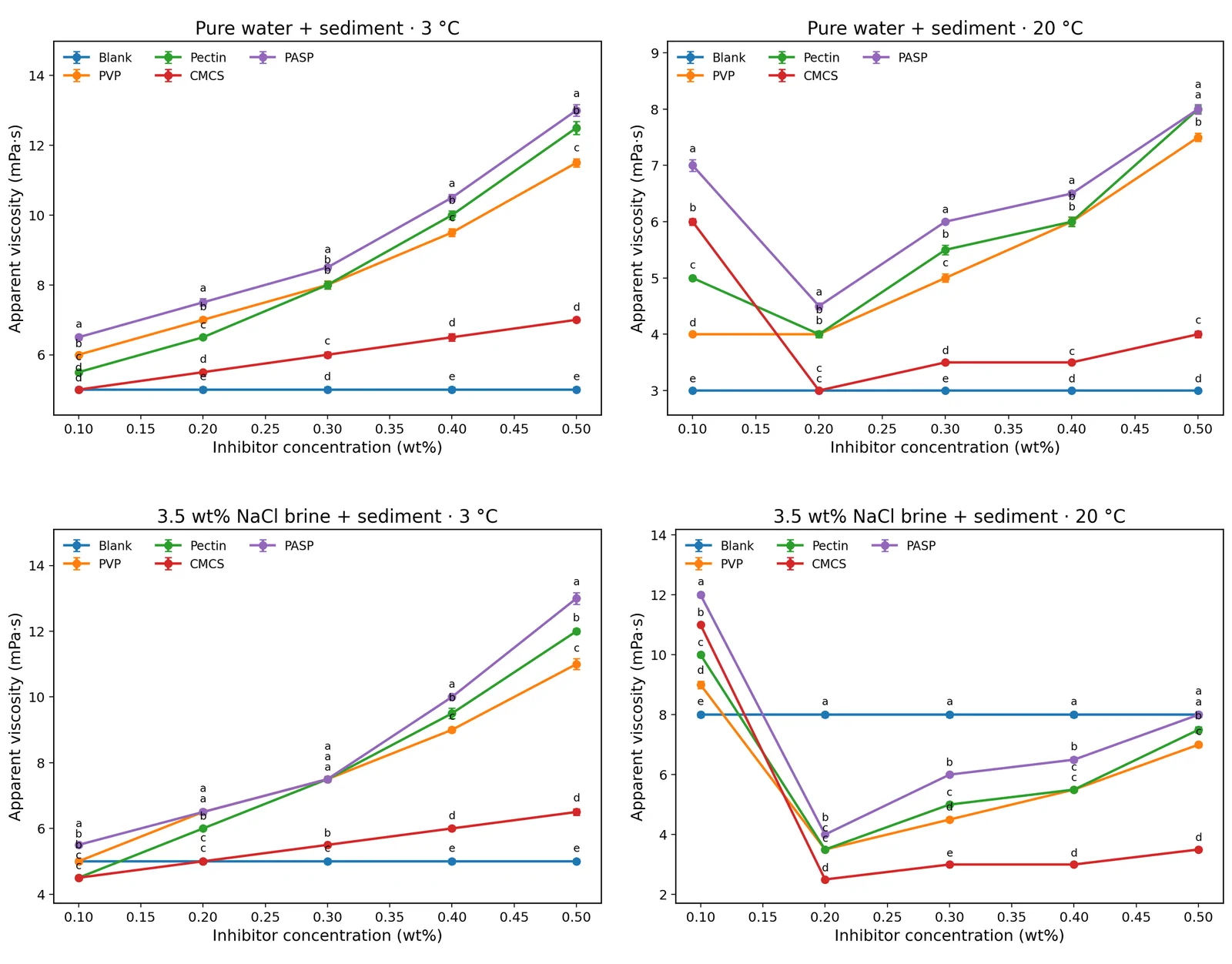
Effects of inhibitor concentration on apparent viscosity at 100 s^−1^ in sediment suspensions. Error bars denote SD (*n* = 3). Within each temperature, concentration, and medium, formulations not sharing a lowercase letter differ significantly at adjusted *p* < 0.05 according to the variance-adaptive procedure in Section 4.2.8.

Apparent viscosity screening at 100 s^−1^ remained below 15 mPa·s for all 0.5 wt% polymer formulations at 3 °C (Figure 12). PASP and pectin gave the highest screening values, while the full flow curves showed substantially larger formulation separation at low shear.

#### 2.4.2. Steady-Shear, Stress Ramp, and Oscillatory Rheological Fingerprints

**Figure 13:**
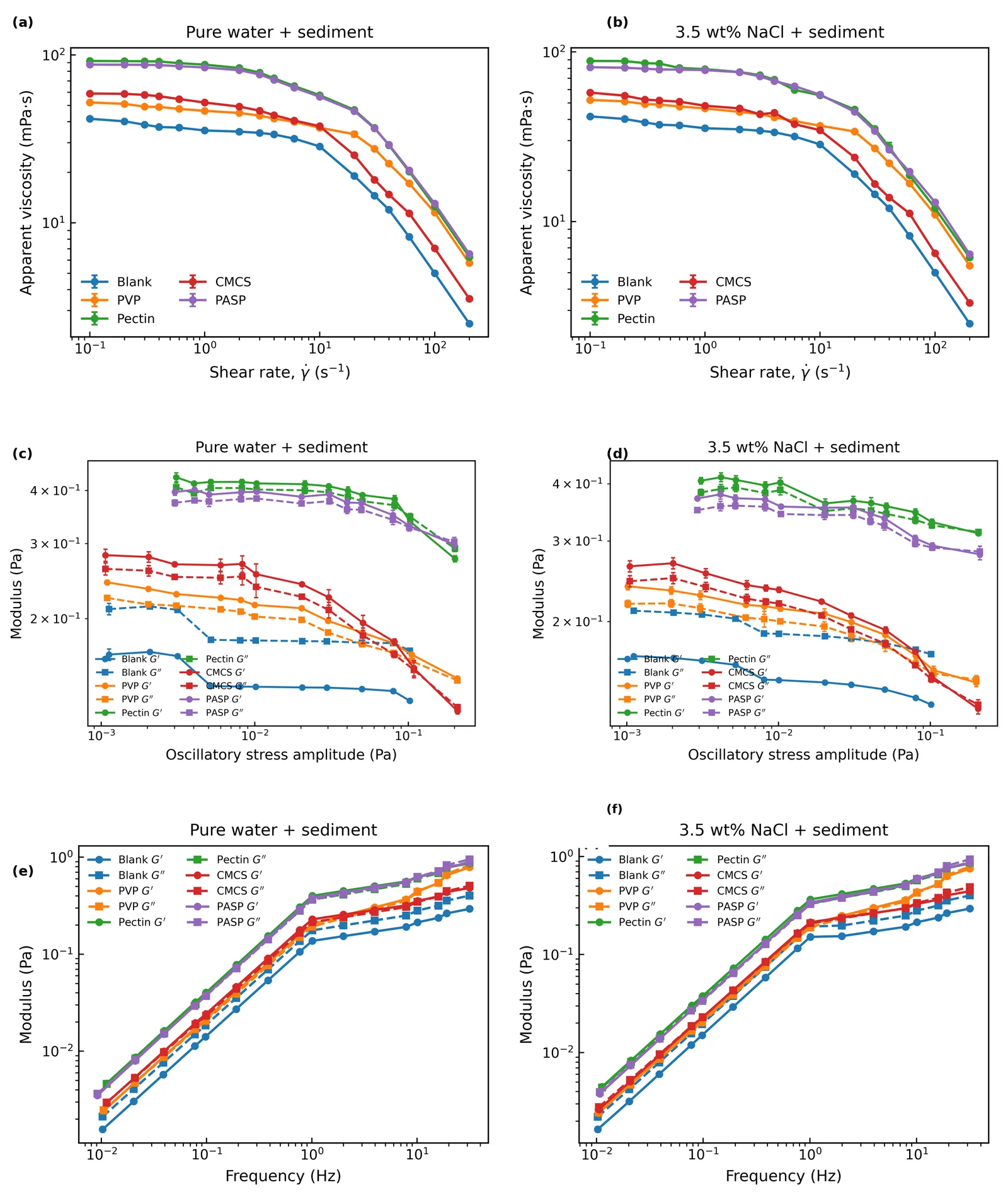
Quantitative rheological characterization of 0.5 wt% formulations at 3 °C in pure water–sediment and 3.5 wt% NaCl brine–sediment media: (**a**,**b**) apparent viscosity as a function of shear rate; (**c**,**d**) stress-controlled oscillatory amplitude sweeps at 1 Hz showing G′ and G″ as functions of oscillatory stress amplitude; and (**e**,**f**) frequency sweeps performed within the linear viscoelastic range. Curves show mean values and error bars denote SD where visible (*n* = 3).

All 0.5 wt% sediment-containing formulations were shear-thinning over 0.1–200 s^−1^ (Figure 13a,b). PASP changed little after NaCl addition, whereas CMCS showed the largest decrease among the polymers at 100 s^−1^.

At 1 Hz, the polymer-containing suspensions were weakly elastic-dominant at low stress, while the blanks were viscous-dominant from the outset (Figure 13c,d; Table 3). Pectin showed the largest salinity shift in oscillatory flow point, from 0.0924 ± 0.0019 to 0.1779 ± 0.0020 Pa; PVP changed from >0.209 Pa to 0.1559 ± 0.0035 Pa. These crossovers are reported as oscillatory flow points because yielding is protocol-dependent [26].

**Figure 14:**
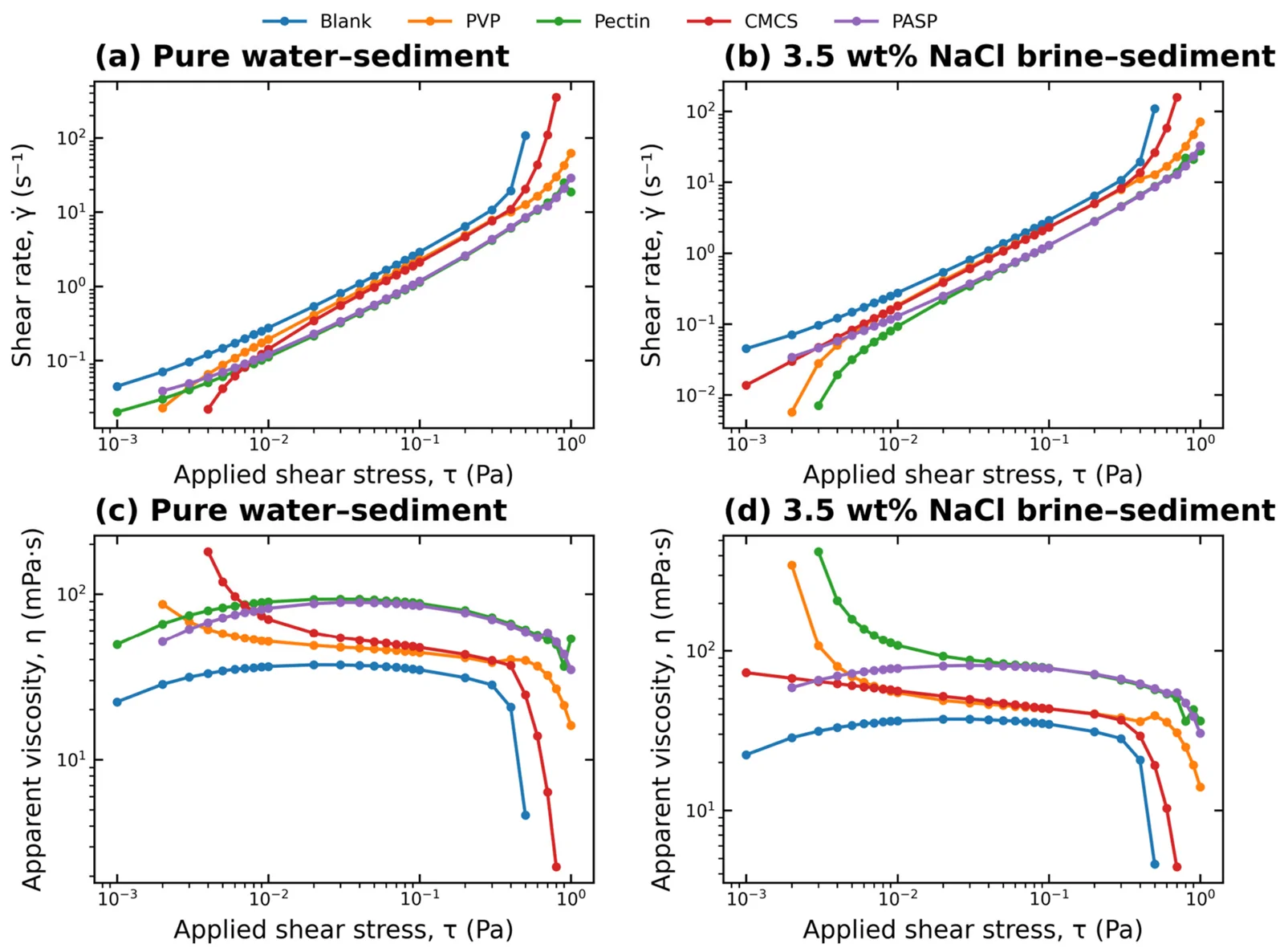
Controlled-shear-stress (CSS) ramp responses of 0.5 wt% formulations at 3 °C: (**a**,**b**) shear rate as a function of applied stress and (**c**,**d**) apparent viscosity as a function of applied stress in pure water–sediment and 3.5 wt% NaCl brine–sediment media, respectively. Curves show means from triplicate datasets (*n* = 3).

CSS ramps showed continuous increases in shear rate with applied stress across all formulations (Figure 14), a response consistent with progressive yielding. Several apparent viscosity maxima occurred at the lower measurement boundary, while the full stress–response curves remained continuous. The ramp behavior is consistent with weak, deformable structuring rather than an abrupt common transition.

Frequency sweeps reinforced this picture: pectin had the highest G′ and lowest tan δ near 1 Hz, whereas tan δ remained close to unity and frequency-dependent crossovers persisted for the polymer-containing suspensions (Figure 13e,f; Table 3).

#### 2.4.3. Structural Recovery and Structure–Performance Relationship

**Figure 15:**
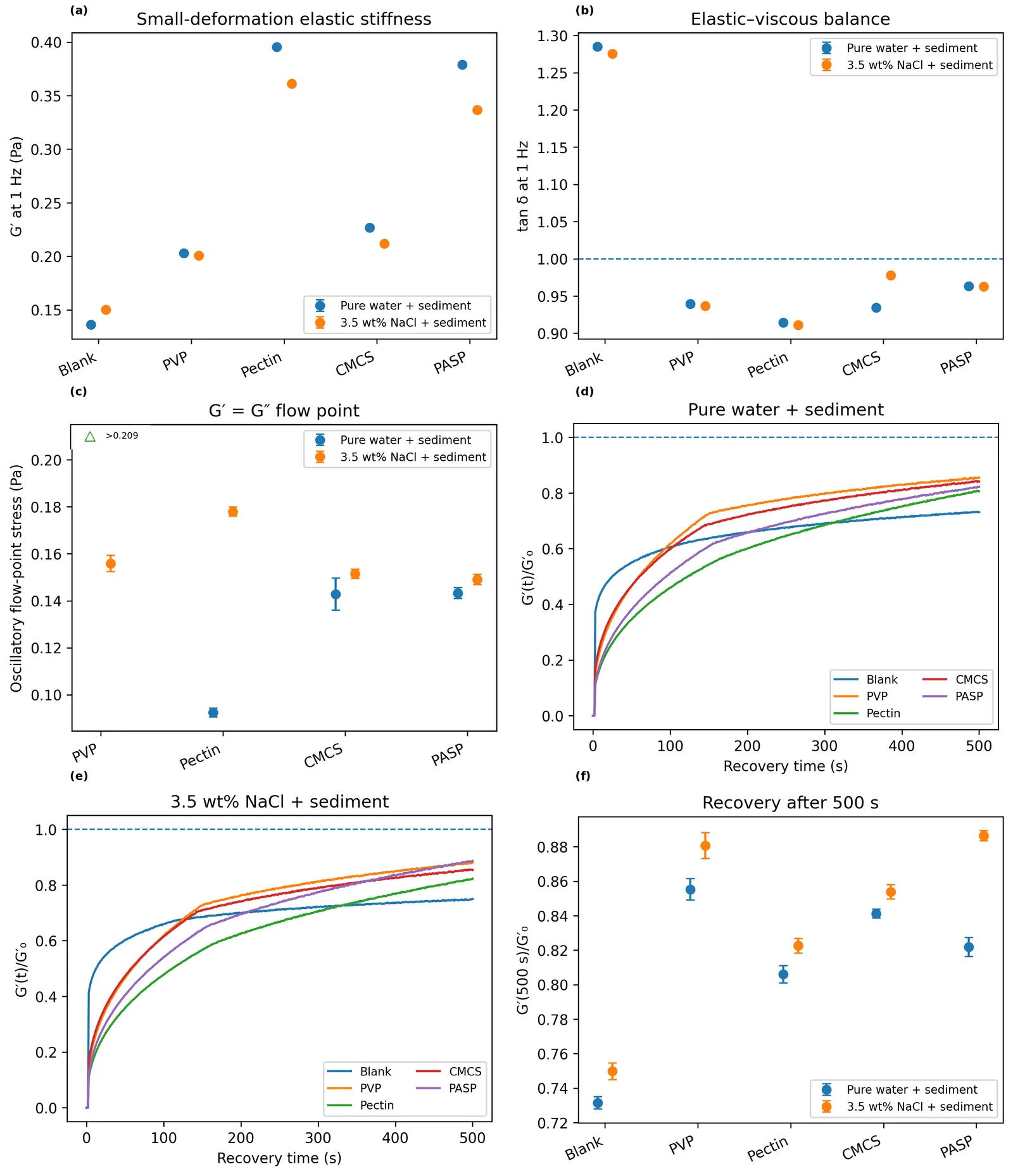
Rheological summary and structural recovery of 0.5 wt% formulations at 3 °C: (**a**) storage modulus G′ near 1 Hz; (**b**) tan δ near 1 Hz; (**c**) oscillatory flow-point stress defined by G′ = G″ from the stress-controlled amplitude sweep (the pure water PVP flow point was not reached and is shown as >0.209 Pa); (**d**,**e**) normalized storage modulus recovery curves in pure water–sediment and 3.5 wt% NaCl brine–sediment, respectively; and (**f**) recovered G′(500 s)/G′0. Error bars denote SD (*n* = 3).

After 100 s^−1^ shear disruption, normalized G′ recovered over 500 s in all formulations (Figure 15d,e; Table 3). Pectin recovered less than the other polymers despite its high initial G′, whereas PASP reached the highest polymer recovery in brine–sediment (0.8864 ± 0.0029), separating elastic stiffness from post-shear rebuilding.

No single rheological descriptor reproduced the KHI ranking. Pectin showed the strongest small-deformation elasticity and the highest resolved brine oscillatory flow point, whereas PASP retained the most favorable multimetric KHI response. Thus, elastic stiffness, progressive yielding, oscillatory flow transition, recovery, and KHI performance are only partially coupled.

### 2.5. Structure–Performance Interpretation and Practical Scope

The pectin–PASP contrast defines the central structure–performance result. Pectin (~200 kDa) produced stronger small-deformation structuring, consistent with its high molecular weight and hydroxyl/carboxyl functionality. Molecular weight can also influence hydrodynamic volume, chain relaxation/entanglement, and interfacial coverage, although these effects cannot be separated from polymer chemistry in the present cross-polymer comparison. PASP (~5 kDa) combined dense carboxylate/amide functionality with greater molecular accessibility and retained the most favorable multimetric KHI response. Stronger macroscopic structuring therefore did not translate directly into stronger hydrate inhibition.

Literature comparisons reinforce the protocol dependence of KHI ranking. Pectin has shown both strong anti-nucleation behavior in induction-based tests [14] and more limited performance in seeded crystal growth tests [13], while homo-poly(aspartic acid) has ranked below more amphiphilic poly(amino acid)s in slow-cooling experiments [23]. Under the present fixed 7.0 MPa and 3 °C protocol, pectin and PASP both strongly delayed hydrate formation, but their rheological fingerprints remained distinct. Differences in pressure, subcooling, polymer source/molecular weight, seeding history, and endpoint definition preclude direct numerical ranking across studies.

NaCl altered the rheological response in a polymer-specific manner, most clearly for the pectin oscillatory flow point. In natural brines, Ca^2+^ and Mg^2+^ can modify carboxyl-containing polymers through charge screening and ion bridging, while SO_4_^2−^ and HCO_3_^−^ can alter ionic speciation and competitive hydration; Ca^2+^-induced pectin structuring illustrates this ion-specific sensitivity [31,32,33,34]. The present single-salt matrix therefore serves as a controlled baseline for multicomponent brine, repeated-cycle, compatibility, and molecular-scale validation.

## 3. Conclusions

### 3.1. Directly Supported Findings

Across the four media, PASP retained the most favorable multimetric KHI response, while pectin showed comparatively stable performance. All sediment-containing formulations were shear-thinning and weakly viscoelastic; pectin had the highest G′, whereas PASP remained the strongest KHI performer despite lower elastic stiffness.

CSS ramps, oscillatory sweeps, and modulus recovery consistently showed weak, deformable, and partially recoverable sediment-associated structuring. Their different rankings demonstrate that elastic stiffness, stress-dependent yielding behavior, oscillatory flow transition, recovery, and KHI performance are distinct material responses.

### 3.2. Proposed Interpretation and Future Validation

The central finding is the partial decoupling of KHI performance from macroscopic rheological structuring. Pectin favors stronger chain/particle structuring, whereas PASP achieves stronger inhibition through a different balance consistent with hydration, electrostatics, and molecular/interfacial accessibility.

The controlled NaCl–sediment matrix provides a baseline for multicomponent brine, higher-pressure dynamic, repeated-cycle, compatibility, environmental, techno-economic, and molecular-scale validation.

## 4. Materials and Methods

### 4.1. Materials and Instruments

The materials used in this study included CH4 gas (purity > 99.99%), PVP (approximately 40,000 Da, purity > 99%), pectin (approximately 200,000 Da, purity ≥ 98%), carboxymethyl chitosan (CMCS, approximately 5000 Da, purity > 98%), and polyaspartic acid (PASP, approximately 5000 Da, purity > 98%). Pectin, CMCS, and PASP were evaluated as hydrated polymeric KHI candidates. The sediment sample was collected from the South China Sea seabed. Whole-rock XRD quantified 66.8 wt% non-clay minerals and 33.2 wt% clay minerals. The non-clay fraction consisted of quartz (38.2 wt%), K-feldspar (0.8 wt%), plagioclase (8.7 wt%), calcite (14.2 wt%), dolomite (2.3 wt%), halite (1.1 wt%), and pyrite (1.5 wt%). Within the clay mineral fraction, illite, kaolinite, and chlorite accounted for 56%, 15%, and 29%, respectively. Two particle size determinations gave median diameters of 8.81 and 8.68 μm (Table 4). Organic matter content, specific surface area, and initial moisture content were outside the characterization set used for this batch. Deionized water with a resistivity of 18.25 MΩ·cm was prepared in the laboratory. A 3.5 wt% NaCl solution was used as a simplified single-salt brine and was not intended to reproduce natural seawater.

**Table 4:** Mineralogical composition and particle size characteristics of the tested sediment.

Parameter	Value	Parameter	Value
Total non-clay minerals	66.8 wt%	Total clay minerals	33.2 wt%
Quartz	38.2 wt%	Illite	56% of clay fraction
K-feldspar	0.8 wt%	Kaolinite	15% of clay fraction
Plagioclase	8.7 wt%	Chlorite	29% of clay fraction
Calcite	14.2 wt%	D50, determination 1	8.81 μm
Dolomite	2.3 wt%	D50, determination 2	8.68 μm
Halite	1.1 wt%	Pyrite	1.5 wt%

Note: Whole-rock XRD gave 66.8 wt% non-clay minerals and 33.2 wt% clay minerals. Illite, kaolinite, and chlorite percentages are relative to the clay mineral fraction. Two particle size determinations yielded median diameters of 8.81 and 8.68 μm.

**Figure 16:**
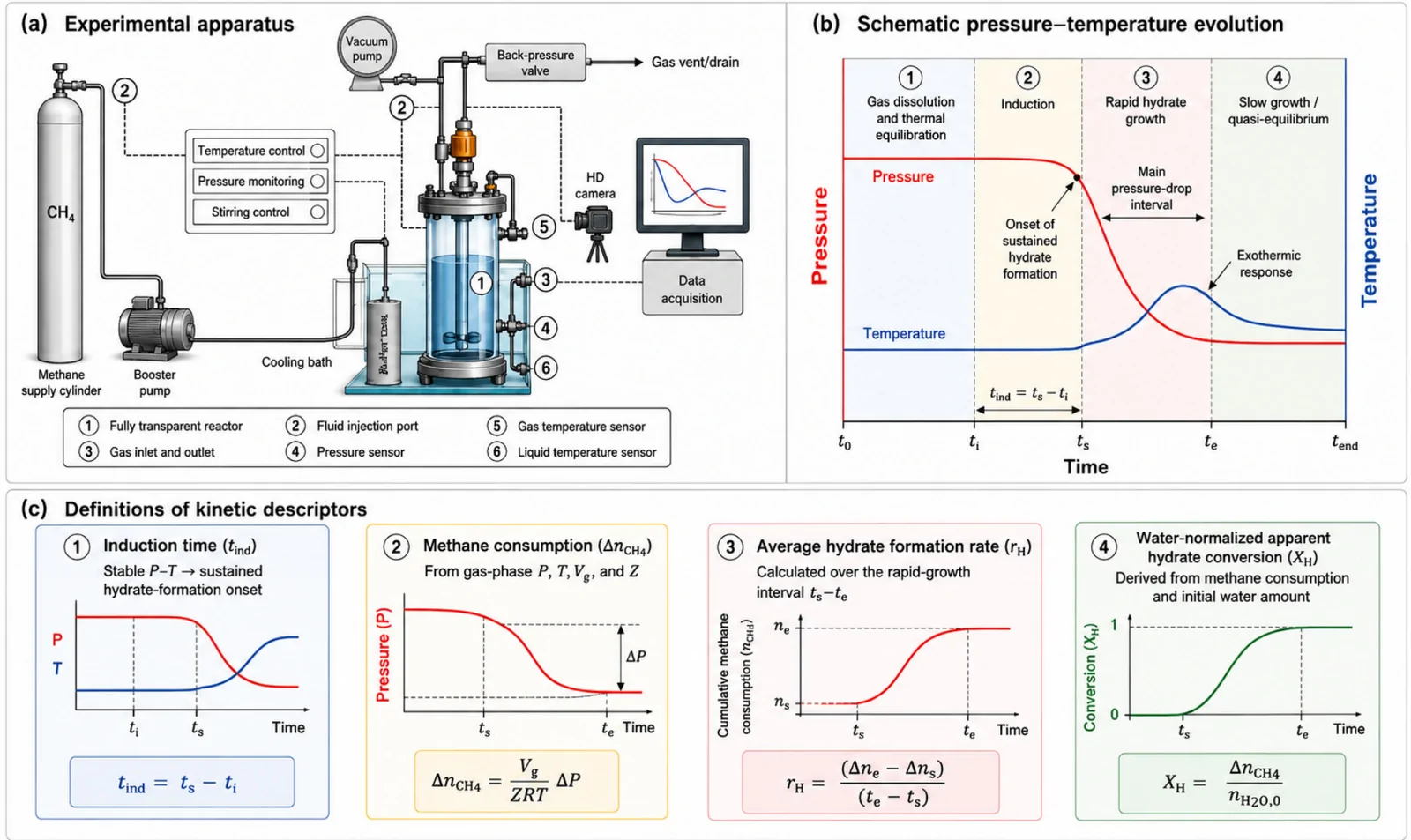
Experimental apparatus, schematic hydrate formation stages, and definitions of kinetic descriptors.

Hydrate inhibition tests were carried out using a fully transparent hydrate formation and dissociation apparatus (Figure 16). Figure 16 also illustrates how pressure–temperature profiles were used to define the hydrate formation stages and extract induction time, methane consumption, average formation rate, and apparent conversion. The transparent reactor had an inner diameter of 40 mm and a height of 100 mm and was equipped with a pressure transducer (accuracy: ±0.01 MPa), a Pt100 temperature probe (accuracy: ±0.1 K), and a data acquisition system. Pressure and temperature were recorded every 5 s. Raman spectra were acquired using a LabRAM HR Evolution confocal Raman spectrometer (HORIBA France SAS, Palaiseau, France). Freeze-dried samples were characterized using a ZEISS Merlin compact field-emission scanning electron microscope (Carl Zeiss Microscopy GmbH, Jena, Germany) coupled with a Bruker XFlash 6|30 energy-dispersive X-ray spectroscopy detector (Bruker Nano GmbH, Berlin, Germany).

### 4.2. Methods

#### 4.2.1. Hydrate Inhibition Tests

Hydrate inhibition was evaluated using a constant-volume, constant-temperature protocol [35,36,37]. The experimental temperature was set to 3.0 °C according to the mudline temperature of the Shenhu area in the South China Sea [38], and the initial pressure was set to 7.0 MPa. Briefly, the transparent reactor was cleaned with deionized water and leak-tested with N_2_. Then, 30 mL of test solution was added, the stirring rate was set to 300 rpm, and the circulating bath was stabilized at 3.0 °C. After thermal equilibration, the reactor was evacuated for 30 min, charged with CH_4_ to 7.0 MPa, and stirred at 600 rpm. Pressure and temperature were recorded until the end of each experiment, while hydrate formation was monitored using a high-definition camera. Each test was repeated three times. Unless otherwise stated, data are reported as mean ± SD, and error bars represent SD (*n* = 3).

Methane consumption, average formation rate, and water-normalized apparent hydrate conversion were used to evaluate kinetic inhibition performance. Because the first cage-like precursor cannot be directly observed in the pressure reactor, an operational induction time was used in this study. It was defined as the time interval from the point at which methane charging reached stable temperature and pressure to the onset of a sustained pressure drop and/or a detectable exothermic temperature signal. Methane consumption was used to quantify the conversion of gas-phase methane into the hydrate phase. Because the tests were conducted in a constant-volume reactor, methane consumption was calculated [39,40] from changes in gas-phase pressure, temperature, and compressibility factor:

(1) $${∆n}_{{CH}_{4,t}}=n_{0}-n_{t}=\frac{P_{0}V_{g}}{Z_{0}RT_{0}}-\frac{P_{t}V_{g}}{Z_{t}RT_{t}}$$

where ΔnCH_4,t_ is the methane consumption at time t (mol); n_0_ and n_t_ are the gas-phase methane amounts at the initial state and at time t (mol), respectively; P_0_ and P_t_ are the corresponding gas-phase pressures (Pa); T_0_ and T_t_ are the corresponding gas-phase temperatures (K); V_g_ is the gas volume (m^3^); R is the gas constant, 8.31441 J/(mol·K); and Z_0_ and Z_t_ are the methane compressibility factors at the initial state and at time t, respectively. The compressibility factor was calculated using the Peng–Robinson equation of state; the same calculation procedure was used throughout the data processing.

The average formation rate was used to describe the mean methane consumption rate during the rapid hydrate growth stage:

(2) $$r_{H}=\frac{{∆n}_{{CH}_{4,e}}-{∆n}_{{CH}_{4,s}}}{t_{e}-t_{s}}$$

where r_H_ is the average hydrate formation rate (mol/min); ΔnCH_4,s_ and ΔnCH_4,e_ are the methane consumptions at the start and end of the rapid growth stage (mol), respectively; and t_s_ and t_e_ are the start and end times of this stage (min), respectively.

The water-normalized apparent hydrate conversion was used to evaluate the extent of water conversion corresponding to methane consumption:

(3) $$X_{H}=\frac{6.1∆n_{{CH}_{4,e}}}{n_{w,0}}\times 100\%$$

where X_H_ is the apparent hydrate conversion (%), ΔnCH_4,e_ is the methane consumption at the end of hydrate formation (mol), n_w,0_ is the initial amount of added water (mol), and 6.1 is the approximate hydration number of methane hydrate used to convert methane consumption into the corresponding water consumption. This indicator is an apparent conversion normalized by the initial water amount; it does not correct for dissolved methane, unreacted water, or spatial heterogeneity within the reactor.

#### 4.2.2. Raman Spectroscopy

Raman spectra were collected using a LabRAM HR Evolution confocal Raman spectrometer (HORIBA France SAS, Palaiseau, France) with a 532 nm excitation laser, a power of 50.0 mW, an integration time of 10 s, a spectral range of 100–4000 cm^−1^, and a resolution of 0.5 cm^−1^. All samples were measured at room temperature in triplicate. After baseline correction, the O-H stretching region was fitted with Gaussian peaks. The relative water structure intensity C was defined as the ratio of the integrated area of the low-wavenumber strong-hydrogen-bond peak (*I_L_*, 3220 ± 20 cm^−1^) to that of the high-wavenumber weak-hydrogen-bond peak (*I_H_*, 3430 ± 20 cm^−1^). A lower C value was used as a comparative descriptor of reduced strongly hydrogen-bonded, ordered water structures; it should not be interpreted as a direct measurement of hydrate cage precursors:

(4) $$C=\frac{I_{L}}{I_{H}}$$

where *I_L_* and *I_H_* are the integrated areas of the fitted peaks near 3220 ± 20 cm^−1^ and 3430 ± 20 cm^−1^, respectively.

#### 4.2.3. Capillary Suction Time

Capillary suction time (CST; Triton Electronics Ltd., Dunmow, UK) was used as an operational descriptor of liquid migration through a standard filter paper geometry. Before testing, each sample was equilibrated at 3 °C for 30 min. A 5.0 mL aliquot was injected into a test cup equipped with a stainless-steel cylinder (inner diameter: 18 mm) and Whatman No. 17 standard chromatography paper. The time required for the liquid front to reach a fixed calibrated distance was recorded. A longer CST indicates slower water migration and was interpreted as a transport descriptor influenced by polymer hydration, viscosity, and particle association. Each measurement was performed in triplicate.

#### 4.2.4. SEM/EDS Mapping Analysis

SEM/EDS mapping was used to observe the mesoscopic morphology and relative surface elemental composition of freeze-dried PVP, pectin, CMCS, PASP, and their sediment suspensions. The test solution or sediment suspension was loaded into an API filter-loss cell, pressurized with N2 to 0.69 MPa, and filtered for 15 min. The resulting filter cake was removed, and a relatively flat region was selected for freeze-drying. The freeze-dried sample was fixed on conductive tape, sputter-coated, and analyzed by SEM imaging and EDS mapping. SEM images were collected using a ZEISS Merlin compact field emission scanning electron microscope (Carl Zeiss Microscopy GmbH, Jena, Germany) at 5.0 kV and a working distance of 9.4 mm with a secondary electron detector. EDS analysis used a Bruker XFlash 6|30 detector (Bruker Nano GmbH, Berlin, Germany), a primary electron energy of 15 keV, and Bruker Esprit 2.0 software. Representative flat filter-cake surfaces were selected for mapping. Because the samples were filtered and freeze-dried, the results were interpreted as ex situ morphology and detected surface composition.

#### 4.2.5. Zeta Potential Measurements

Zeta potential was measured using a Zetasizer Nano ZS instrument (Malvern Panalytical Ltd., Malvern, UK) to evaluate how the inhibitors affected the surface charge and suspension stability of sediment particles. The suspension was ultrasonically dispersed for 5 min at 100 W. An aliquot was transferred into a DTS1070 folded capillary cell and equilibrated at 3 °C or 20 °C for 120 s before measurement. Each sample was measured in triplicate.

#### 4.2.6. Low-Temperature Apparent Viscosity Screening

Apparent viscosity screening was performed using an MCR302 rotational rheometer (Anton Paar GmbH, Graz, Austria) equipped with a PP50 parallel-plate geometry (diameter: 50 mm). Before testing, the sample was ultrasonically dispersed for 5 min at 100 W. The plate gap was 1.0 mm. Samples were equilibrated at 3 or 20 °C for 120 s and measured at a constant shear rate of 100 s^−1^. These measurements covered multiple inhibitor concentrations and two temperatures and were retained as a broad bulk-thickening screen. Each measurement was performed in triplicate. The 0.5 wt%, 3 °C sediment-containing formulations were subsequently subjected to the quantitative rheological protocol described below.

#### 4.2.7. Quantitative Rheological Characterization

Quantitative rheology was conducted at 3 °C using an Anton Paar MCR302 with PP50 geometry and a 1.0 mm gap, following a structured-suspension framework adapted from Shakeel et al. [25] Blank and 0.5 wt% PVP, pectin, CMCS, and PASP formulations were tested in pure water–sediment and 3.5 wt% NaCl brine–sediment. Steady-shear flow curves covered 0.1–200 s^−1^. The rheometer’s standard instrument-controlled CSS ramp procedure was applied stepwise over formulation-specific windows within approximately 0.001–1.0 Pa (blank, 0.001–0.5 Pa; PVP/PASP, 0.002–1.0 Pa; pectin, 0.001–1.0 Pa in pure water and 0.003–1.0 Pa in brine; CMCS, 0.004–0.8 Pa in pure water and 0.001–0.7 Pa in brine). Apparent viscosity and shear rate were recorded against applied stress; first-step viscosity maxima were treated as lower-boundary limited. Oscillatory amplitude sweeps were performed at 1 Hz, and G′ = G″ flow points were obtained for each replicate by linear interpolation of ΔG = G′ − G″ between adjacent bracketing stresses; no extrapolation was applied when no crossover occurred. Frequency sweeps were performed at 0.01% strain over approximately 0.01–31.6 Hz. Structural recovery was measured after 500 s at 100 s^−1^ and expressed as G′(t)/G′0 and G′(500 s)/G′0. CSS ramps were interpreted from the stress–shear-rate and viscosity–stress evolution as continuous stress-dependent flow consistent with progressive yielding, while G′ = G″ was retained as an oscillatory flow-point descriptor [26]. Constrained Herschel–Bulkley fits [41] of the independent steady-flow curves converged to negligible yield terms, consistent with the progressive-flow response. All rheological datasets were obtained in triplicate.

#### 4.2.8. Normalized Performance Index and Statistical Data Treatment

Independent measurements were performed in triplicate and are reported as mean ± SD. Formulation effects were evaluated separately within each medium and concentration at α = 0.05. Because *n* = 3 per group provides little power for formal normality testing, normality tests were not used as the decision rule for selecting the inferential test. Variance homogeneity was assessed using a median-centered Levene test. When homogeneity was supported (*p* ≥ 0.05), conventional one-way ANOVA followed by Tukey’s honestly significant difference (HSD) test was used; when variance heterogeneity was indicated (*p* < 0.05), Welch’s ANOVA followed by Games–Howell pairwise comparisons was used. Adjusted *p* < 0.05 was considered statistically significant. Where shown, lowercase letter groupings indicate the post hoc results within the same medium and concentration; groups not sharing a letter differ significantly. Statistical inference is condition-specific and is not interpreted as a universal material ranking. For exploratory integration, descriptors were converted into dimensionless scores by min–max normalization within each medium. Induction time and absolute zeta potential (|zeta|) were treated as positive indicators, whereas methane consumption, average formation rate, apparent conversion, and apparent viscosity were treated as negative indicators. Available scores were averaged with equal weighting. Equal weighting is an explicit screening assumption rather than an established engineering priority, and the resulting NPI should not be interpreted as a thermodynamic, causal, or field performance index.

## Figures and Tables

**Table 1:** Exploratory equal-weight normalized performance index of PVP, pectin, CMCS, and PASP within the tested media.

NPI Set	Medium	PVP	Pectin	CMCS	PASP
Common descriptors	Pure water	0.50	0.52	0.25	0.90
Common descriptors	Pure water + sediment	0.32	0.48	0.00	1.00
Common descriptors	3.5 wt% NaCl	0.43	0.65	0.00	1.00
Common descriptors	3.5 wt% NaCl + sediment	0.33	0.65	0.00	1.00
Sediment-specific descriptors	Pure water + sediment	0.26	0.44	0.20	0.83
Sediment-specific descriptors	3.5 wt% NaCl + sediment	0.27	0.58	0.18	0.83

Note: The common-descriptor NPI used induction time, methane consumption, average formation rate, and apparent conversion at 0.5 wt%. The sediment-specific NPI additionally included absolute zeta potential and apparent viscosity. Higher values indicate a more favorable equal-weight screening score within the same descriptor set; no universal engineering weighting is implied.

**Table 2:** Relative elemental contents detected by EDS in representative inhibitor–sediment filter-cake regions.

Element	Sediment	Sediment + 0.5 wt% PVP	Sediment + 0.5 wt% Pectin	Sediment + 0.5 wt% CMCS	Sediment + 0.5 wt% PASP
C	10.89	15.28	35.35	15.83	26.68
O	51.32	57.12	44.41	57.32	38.19
Na	0.49	1.09	3.32	1.12	11.80
Al	7.59	5.24	2.79	5.82	3.00
Si	16.26	14.80	9.09	13.17	9.45
Cl	0	0.32	2.16	0.33	8.63
Ca	13.45	6.13	2.88	6.41	2.25

**Table 3:** Quantitative rheological descriptors for 0.5 wt% sediment-containing formulations at 3 °C (mean ± SD, *n* = 3). Blank suspensions were viscous-dominant from the start of the amplitude sweep and therefore have no G′ = G″ flow point. Pure-water PVP remained elastic-dominant to the maximum tested stress and is reported as >0.209 Pa without extrapolation.

Medium	Formulation	η100 (mPa·s)	G′ at 1 Hz (Pa)	tan δ at 1 Hz	Oscillatory Flow-Point Stress (Pa)	G′(500 s)/G′0
PW–sediment	Blank	5.027 ± 0.021	0.1363 ± 0.0005	1.284846 ± 0.000001	—	0.7314 ± 0.0036
PW–sediment	PVP	11.477 ± 0.058	0.2029 ± 0.0003	0.939471 ± 0.000001	>0.209	0.8553 ± 0.0061
PW–sediment	Pectin	12.546 ± 0.063	0.3954 ± 0.0003	0.914206 ± 0.000001	0.0924 ± 0.0019	0.8060 ± 0.0051
PW–sediment	CMCS	7.047 ± 0.032	0.2268 ± 0.0008	0.934326 ± 0.000001	0.1428 ± 0.0068	0.8412 ± 0.0026
PW–sediment	PASP	13.052 ± 0.051	0.3790 ± 0.0012	0.963457 ± 0.000001	0.1432 ± 0.0023	0.8218 ± 0.0055
NaCl–sediment	Blank	5.007 ± 0.014	0.1504 ± 0.0005	1.275491 ± 0.000008	—	0.7498 ± 0.0049
NaCl–sediment	PVP	10.963 ± 0.059	0.2009 ± 0.0006	0.936833 ± 0.000001	0.1559 ± 0.0035	0.8806 ± 0.0075
NaCl–sediment	Pectin	11.991 ± 0.041	0.3611 ± 0.0016	0.910980 ± 0.000001	0.1779 ± 0.0020	0.8225 ± 0.0042
NaCl–sediment	CMCS	6.509 ± 0.024	0.2118 ± 0.0006	0.977701 ± 0.000001	0.1515 ± 0.0020	0.8538 ± 0.0042
NaCl–sediment	PASP	13.068 ± 0.063	0.3368 ± 0.0012	0.962984 ± 0.000001	0.1490 ± 0.0022	0.8864 ± 0.0029

## Data Availability

The data supporting the findings of this study are available from the corresponding author upon reasonable request.

## References

[1] Sloan E.D. (2003). Fundamental principles and applications of natural gas hydrates. Nature.

[2] Hassanpouryouzband A., Joonaki E., Vasheghani Farahani M., Takeya S., Ruppel C., Yang J., English N.J., Schicks J.M., Edlmann K., Mehrabian H. (2020). Gas hydrates in sustainable chemistry. Chem. Soc. Rev..

[3] Aman Z.M. (2021). Hydrate Risk Management in Gas Transmission Lines. Energy Fuels.

[4] Ninomiya S., Konno Y., Wada R., Sato T., Hajohta H., Shuto C., Muraoka M., Yamamoto Y., Suzuki K. (2024). Dry Flow Technology as Flow Assurance in Methane Hydrate Development. Energy Fuels.

[5] Effendi A.D., Jiaqi L., Sia C.W., Jasamai M., Sulaimon A.A. (2022). Polysaccharides from *Tamarindus indica* L. as natural kinetic hydrates inhibitor at high subcooling environment. J. Pet. Explor. Prod. Technol..

[6] Kamal M.S., Hussein I.A., Sultan A.S., von Solms N. (2016). Application of various water soluble polymers in gas hydrate inhibition. Renew. Sustain. Energy Rev..

[7] Lim J., Kim E., Seo Y. (2016). Dual inhibition effects of diamines on the formation of methane gas hydrate and their significance for natural gas production and transportation. Energy Convers. Manag..

[8] Li B., Lu Y.-Y., Li Y.-L. (2022). A Review of Natural Gas Hydrate Formation with Amino Acids. J. Mar. Sci. Eng..

[9] Kelland M.A., Zhang Q. (2026). Review Series on Factors Affecting Kinetic Hydrate Inhibitor (KHI) Performance. Part 1: Designing the KHI Polymer. Energy Fuels.

[10] Navaneetha Kannan S., Delgado-Linares J.G., Makogon T.Y., Koh C.A. (2024). Synergistic effect of Kinetic Hydrate Inhibitor (KHI) and Monoethylene Glycol (MEG) in gas hydrate management. Fuel.

[11] Zhang Q., Kelland M.A., Ajiro H. (2020). Polyvinylsulfonamides as Kinetic Hydrate Inhibitors. Energy Fuels.

[12] Kelland M.A. (2018). A Review of Kinetic Hydrate Inhibitors from an Environmental Perspective. Energy Fuels.

[13] Aminnaji M., Anderson R., Jarrahian K., Tohidi B. (2022). Natural Pectin and Commercial Luvicap-Bio as Green Kinetic Hydrate Inhibitors: A Comparative Evaluation by Crystal Growth Inhibition Methods. Energy Fuels.

[14] Xu S., Fan S., Fang S., Lang X., Wang Y., Chen J. (2016). Pectin as an Extraordinary Natural Kinetic Hydrate Inhibitor. Sci. Rep..

[15] Zheng H., Wei N., Guo B., Wang J. (2025). Multistage inhibition mechanism of L-aspartic acid polymerized hydrate inhibitor: A combined study of molecular simulation and experimental investigation. J. Mol. Liq..

[16] Mu H., Liu Y., Ding L., Wang W., Lv X., Yu Y., Ma Q., Wang C., Li X., Zhou S. (2026). A molecular study of synergistic inhibition of methane hydrate growth with glycine-based peptides and PVCap. Fuel.

[17] Zhang Y., Li Z., Yang X., Li T. (2022). Synthesis of Chitosan Derivatives and Their Inhibition Effects on Methane Hydrates. Energies.

[18] Chen Z., Farhadian A., Shaabani A., Semenov M.E., Chen C. (2024). Molecular and experimental insights into the inhibition effects of chitosan biguanidine on the kinetic and agglomeration of gas hydrates. Fuel.

[19] Jia Y., Zhao Y., Li M., Zhang L., Liu Y., Dong H., Zhao J., Yang L., Song Y. (2022). Biodegradable Organics as a Multisystem-Compatible Low-Dose Green Kinetic Hydrate Inhibitor. ACS Sustain. Chem. Eng..

[20] Wang J., Sun J., Bian H., Wang Q., Feng Z., Lu C., Ren H., Cheng R., Wang J., Wang R. (2022). Kinetic Hydrate Inhibition of Natural Gels in Complex Sediment Environments. Gels.

[21] Li Z., Zhang Y., Shen Y., Yang X., Li T., Chen G. (2022). The study on the relationship between the molecular structures of chitosan derivatives and their hydrate inhibition performance. J. Mol. Liq..

[22] Singh A., Suri A. (2023). Synergistic Kinetic Hydrate Inhibition of Pectin, PVP, and PVCap with Monoethylene Glycol. Energy Fuels.

[23] Zhang Q., Li Z., Wu G., Tang X., Lu H. (2023). Inhibition effect of novel amphiphilic poly(amino acid)s on methane hydrate. Gas. Sci. Eng..

[24] Liu K., Zi M., Zou X., Yao Y., Wang Y., Yang C., Chen D. (2024). Determining the Critical Time Points for Hydrate Formation in the Presence of Kinetic Hydrate Inhibitors: A Rheological and Kinetic Study. Fuel.

[25] Shakeel A., Kirichek A., Chassagne C. (2021). Rheology and yielding transitions in mixed kaolinite/bentonite suspensions. Appl. Clay Sci..

[26] García-Romero E.L., Daza-Gómez L.-C., Ascanio G., Pérez-Salas K.Y., Aguayo J.P. (2026). The dependence of the yield stress on the rheological method and microstructure in soft materials. Korea-Aust. Rheol. J..

[27] Kitano H., Imai M., Gemmei-Ide M., Takaha K. (2004). Raman spectroscopic study on the structure of water in aqueous solution of zwitterionic surfactants. J. Colloid Interface Sci..

[28] Sa J.-H., Kwak G.-H., Han K., Ahn D., Lee K.-H. (2015). Gas hydrate inhibition by perturbation of liquid water structure. Sci. Rep..

[29] Chen G.W., Lin W.W., Lee D.J. (1996). Capillary suction time (CST) as a measure of sludge dewaterability. Water Sci. Technol..

[30] Agmo Hernández V. (2023). An overview of surface forces and the DLVO theory. ChemTexts.

[31] Wilson I., Patel H., Sreenivasan H., Krishna S. (2024). Performance evaluation of fracturing fluids in the presence of NaCl, CaCl_2_ and KCl as hydrate inhibitors for extremely low temperature stimulation applications. Int. J. Hydrogen Energy.

[32] Liao B., Sun J., Wang J., Lv X., Wang J., Guo J., Lv K., Wang R., Zheng J., Chen Z. (2023). Development of novel natural gas hydrate inhibitor and the synergistic inhibition mechanism with NaCl: Experiments and molecular dynamics simulation. Fuel.

[33] Chremos A., Douglas J.F. (2018). Competitive Solvation Effects in Polyelectrolyte Solutions. Gels and Other Soft Amorphous Solids.

[34] Basak R., Bandyopadhyay R. (2014). Formation and rupture of Ca^2+^-induced pectin biopolymer gels. Soft Matter.

[35] Skovborg P., Ng H.J., Rasmussen P., Mohn U. (1993). Measurement of induction times for the formation of methane and ethane gas hydrates. Chem. Eng. Sci..

[36] Mohammad-Taheri M., Tohidi B., Ghanbari B., Taheri Rizi Z. (2024). Improved industrial induction time-based technique for evaluating kinetic hydrate inhibitors. Orig. Res..

[37] Kvamme B., Aromada S.A., Saeidi N., Hustache-Marmou T., Gjerstad P. (2020). Hydrate Nucleation, Growth, and Induction. ACS Omega.

[38] Liu L., Sun Z., Zhang L., Wu N., Yichao Q., Jiang Z., Geng W., Cao H., Zhang X., Zhai B. (2019). Progress in Global Gas Hydrate Development and Production as a New Energy Resource. Acta Geol. Sin..

[39] Abedi-Farizhendi S., Iranshahi M., Mohammadi A., Manteghian M., Mohammadi A.H. (2019). Kinetic study of methane hydrate formation in the presence of carbon nanostructures. Pet. Sci..

[40] Susilo R., Ripmeester J.A., Englezos P. (2007). Methane conversion rate into structure H hydrate crystals from ice. AIChE J..

[41] Saasen A., Ytrehus J.D. (2020). Viscosity Models for Drilling Fluids—Herschel-Bulkley Parameters and Their Use. Energies.

